# Changes of coagulation function and risk of stroke in patients with COVID‐19

**DOI:** 10.1002/brb3.2185

**Published:** 2021-05-16

**Authors:** Feng Qiu, Yue Wu, Aiqing Zhang, Guojin Xie, Hui Cao, Mingyang Du, Haibo Jiang, Shun Li, Ming Ding

**Affiliations:** ^1^ Cerebrovascular Disease Center Nanjing Brain Hospital Affiliated to Nanjing Medical University Nanjing, Jiangsu Province China; ^2^ Neonatal Medical Center Children’s Hospital of Nanjing Medical University Nanjing, Jiangsu Province China; ^3^ Department of Pediatric Nephrology the Second Affiliated Hospital of Nanjing Medical University Nanjing, Jiangsu Province China; ^4^ Department of Clinical Laboratory Children’s Hospital of Nanjing Medical University Nanjing, Jiangsu Province China; ^5^ Department of Respiratory Medicine Zhongda Hospital School of Medicine Southeast University Nanjing, Jiangsu Province China

**Keywords:** cerebrovascular disease, coagulation function, coronavirus, COVID‐19, respiratory infection, stroke

## Abstract

**Background and purpose:**

COVID‐19 is spreading throughout the whole world as a public health issue. There is a link between the new coronavirus and changes in biochemical indicators, such as coagulation functions. Hypercoagulable state of blood caused by infections may lead to cerebrovascular diseases. More attention should be paid to patients with COVID‐19, especially critically ill individuals with history of cerebrovascular disease who may have high risk of stroke.

**Methods:**

193 patients with COVID‐19 were enrolled in the study. These patients were categorized into nonsevere (143 patients) and severe (50 patients) groups. This study evaluated laboratory tests, including routine blood tests, C‐reactive protein, erythrocyte sedimentation rate, electrolytes, and coagulation functions. Furthermore, neurological function and stroke risks were evaluated in this study.

**Results:**

Compared to the nonsevere group, there were increases in white blood cells, neutrophil count, interleukin‐6, erythrocyte sedimentation rate, and C‐reactive protein in the severe group (*p* < .05). For coagulation functions, parameters like prothrombin time, international normalized ratio, activated partial thromboplastin time, thrombin time, D‐dimer, and fibrin degradation products were increased significantly in the severe group (*p* < .01). Severe patients also demonstrated higher scores on the Framingham stroke risk profile and lower Glasgow scores (*p* < .05). Furthermore, significant associations were noticed between stroke risk and age, blood cell count, neutrophil count, D‐dimmer, and fibrin degradation productions (*p* < .05).

**Conclusions:**

Data suggested that coagulation functions were affected in patients with COVID‐19. Hypercoagulable state in patients may lead to potential high risk of stroke.

## Graphical abstract

The study examined whether there were differences in coagulation functions between critically ill patients with COVID‐19 and nonsevere patients. Whether these changes were associated with neurological function and stroke risks were also examined.

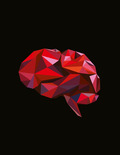

## 1 INTRODUCTION

Since late 2019, a new fatal virus has been spreading throughout the whole world, causing thousands of deaths to date. The World Health Organization (WHO) (World health organization, [Link], 2020) labeled it as coronavirus disease‐2019 (COVID‐19), which is characterized by respiratory system infections that can range from mild to very severe. Cough and fever were the most common symptoms at the onset, diarrhea also occurs in some cases (Guan et al., 2020). This disease demonstrates a high variability of symptoms within individuals, with some patients exhibiting only mild symptoms that include fever, cough, and body malaise, while others may develop severe pneumonia that can result in death without specialist intensive care. It was reported that around 20% of COVID‐19 patients were critically ill patients, and the mortality rate reached around 4.3% to 14.6% (Chen, Zhou, et al., 2020; Guan et al., 2020; Huang et al., 2020; Wang, Hu, et al., 2020). Although the underlying comorbidities such as hypertension, cerebrovascular disease, and diabetes were not obvious among patients, the presence of such medical conditions was more common among critically ill patients (Huang et al., 2020; Wang, Hu, et al., 2020). Previous studies have shown that coronaviruses were closely related to neurological symptoms and acute respiratory tract infections that can raise the risk of cerebrovascular disease and leave patients with an elevated risk of stroke for up to 28 days (Abdelaziz & Waffa, 2020; Warren‐Gash et al., 2018). Despite the established link between other coronaviruses and cerebrovascular diseases, there has been little research concerning the impact of COVID‐19 on risk of stroke, especially in patients with more severe symptoms. Furthermore, previous research has not examined coagulation function to determine stroke risk and provide pre‐therapeutic targets. Given that coagulopathy may be associated with higher mortality in COVID‐19 patients, our study evaluated the coagulation function in patients with COVID‐19 to explore the possible mechanisms of stroke risk in those suffering with this disease.

## 2 METHODS

### 2.1 Study design and subjects

All experimental procedures were approved by the Human Research Ethics Committee of Huangshi Traditional Chinese Hospital, HuBei Province (Huangshi Infectious Disease Hospital) (HSZYPJ‐2020–018–01).

This study was a retrospective study involving data from 193 patients from Huangshi Traditional Chinese Medicine Hospital who were treated for COVID‐19 between January 1st to March 20th, 2020. All patients were diagnosed with the new coronavirus pneumonia and categorized according to the diagnosis and treatment plan (Version 7) (National Health Commission of the People's Republic of China,). In this study, patients were categorized into two groups based on the degree of severity of COVID‐19:143 patients were in the nonsevere group and 50 patients were in the severe group.

### 2.2 Data collection

#### 2.2.1 Demographic and clinical characteristics

Demographic characteristics, including age, gender, and past medical history, were collected from all patients, and laboratory tests were undertaken for each patient at admission. The laboratory data consisted of routine blood tests, platelet count, C‐reactive protein (CRP), erythrocyte sedimentation rate (ESR), and electrolytes.

#### 2.2.2 Coagulation functions

Blood samples were collected at admission and analyzed by an ACL TOP700 Coagulation Analyzer (Werfen Co., Ltd, China) for coagulation assessments, which included prothrombin time (PT), international normalized ratio (INR), activated partial thromboplastin time (APTT), thrombin time (TT), fibrinogen (FIB), fibrin degradation product (FDP), and D‐dimer.

#### 2.2.3 Neurological function and stroke risk evaluation

Assessments of stroke risk and neurological function were made using the Framingham Stroke Risk Profile (Dufouil et al., 2017) and the Glasgow Coma Scale (Jennett et al., 1976). Specifically, the Framingham Stroke Risk Profile (FSRP) was utilized to evaluate stroke risk factors, such as age, gender, systolic blood pressure, left ventricular hypertrophy, smoking status, and the medical history of cardiovascular disease, atrial fibrillation, or diabetes mellitus. All scores were summed to predict the probability of incident stroke in 10 years. The higher the score on this assessment, the higher the likelihood of cerebrovascular disease. The Glasgow Coma Scale (GCS) (Jennett et al., 1976) was completed for each patient at admission and consists of three components; i) eye; ii) verbal; and iii) motor responses. Patients were assessed to judge their level of consciousness and the severity of their disease. The maximum score of 15 points reflects full and clear consciousness, while scores of between 12 to 14, 9 to 11, and less than 8 represent mildly injured, moderately injured, and severely injured, respectively.

### 2.3 Statistical analysis

Differences in variables within two groups were determined by using Student's *t* test. Comparison of different subgroups was performed by One‐way ANOVA or the Kruskal–Wallis test, followed by Tukey's post hoc test for multiple comparisons. Data obtained were shown as Mean ± *SD* (standard deviation) values, while Pearson's and Spearman's rank correlations were used for correlation analysis. Statistical analyses were completed using SPSS (Version 21.0), and a *p*‐value of <.05 was considered as statistically significant.

## 3 RESULTS

### 3.1 Baseline characteristics

A total of 193 hospitalized patients with COVID‐19 were studied. The mean age was 59.7 years (range, 31–88 years) for critically ill patients, which was significantly older than patients in the nonsevere group with a mean age of 53 years (range, 21–85 years) (*p* <.01). No differences were noticed in sex distinction between the two groups. Of the 193 patients, less than half had underlying diseases. Compared to the nonsevere group, severe patients had more comorbidities (29 [20.2%] versus 19 [38%]), including hypertension (16 [11.2%] versus 16 [32%]) and stroke (4 [2.8%] versus 11 [22%]) (*p* <.05). With respect to patients with stroke conditions, all were ischemic cerebrovascular diseases, and no cerebral hemorrhage was observed. Both groups demonstrated similar rate of coronary heart disease (Table 1).

**TABLE 1:** Demographic characteristics of patients with COVID‐19

	Nonsevere (*n* = 143)	Severe (*n* = 50)	*p* Value
Age, mean±*SD*, years	53.0 ± 14.3	59.7 ± 14.6	.006
Gender, male, *n* (%)	66 (53.1%)	24 (48.0%)	.890
Comorbidities	29 (20.2%)	19 (38%)	.032
Hypertension, *n* (%)	16 (11.2%)	16 (32%)	.001
Diabetes, *n* (%)	8 (5.6%)	6 (12%)	.134
CHD, *n* (%)	3 (2.1%)	2 (4%)	.467
Stroke, *n* (%)	4 (2.8%)	11 (22%)	<.001

Abbreviation: CHD, coronary heart disease.

### 3.2 Results of routine laboratory assessments

Numerous differences were noticed in the results of routine laboratory tests between the nonsevere and severe groups. Both white blood cell and neutrophil count increased significantly in the severe group (*p* <.01), while the decline in lymphocyte count for the severe group was also significant (*p* <.01). No significant differences were noticed in monocyte count, red blood cell count, hemoglobin, or platelet count between the two groups (*p* >.05). With respect to interleukin‐6, the severe group exhibited significantly greater values than the nonsevere group (*p* <.05). Biochemical parameters such as serum sodium, potassium, chloride, and uric acid all showed no differences between the two groups (*p* >.05). Erythrocyte sedimentation rate and C‐reactive protein demonstrated significantly higher values in the severe group (*p* <.05) (Table 2).

**TABLE 2:** The routine laboratory tests of patients with COVID‐19

	Nonsevere (*n* = 143)	Severe (*n* = 50)	*p* Value
White blood cell count	4.81 × 10^9^/L	5.45 × 10^9^/L	.002
Neutrophil count	3.16 × 10^9^/L	4.21 × 10^9^/L	.006
Lymphocyte count	1.14 × 10^9^/L	0.79 × 10^9^/L	.005
Monocyte count	0.45 × 10^9^/L	0.37 × 10^9^/L	.852
Red blood cell count	4.37 × 10^12^/L	4.13 × 10^12^/L	.543
Hemoglobin	130g/L	121.9 g/L	.882
Platelet count	182.8 × 10^9^/L	222 × 10^9^/L	.095
CRP	34.3 mg/L	53.6 mg/L	.014
Serum sodium	139.8 mmol/L	138.2 mmol/L	.142
Serum potassium	4.12 mmol/L	4.02 mmol/L	.667
Serum chloride	104.1 mmol/L	102.7 mmol/L	.245
Serum uric acid	258.9 μmol/L	246.3 μmol/L	.797
ESR	59.54 mm/h	61.38 mm/h	.038
Interleukin−6	5.89 pg/ml	15.51 pg/ml	.034

Abbreviations: ESR, Erythrocyte sedimentation rate; CRP, C‐reactive protein.

### 3.3 Results of coagulation functions

Parameters of coagulation function were evaluated between the two groups (Table 3). Compared to the nonsevere group, significant increases in most parameters were observed in the severe group (*p* <.001), including PT, APTT, TT, INR, D‐dimer, and FDP. Only FIB showed no changes between groups (*p* >.05).

**TABLE 3:** Coagulation functions of patients with COVID‐19

	Nonsevere (*n* = 143)	Severe (*n* = 50)	*P* Value
PT	11.7s	12.9s	<.001
INR	0.93	1.03	<.001
APTT	38.6s	41.1s	<.001
TT	12.5s	19.4s	<.001
FIB	4.63 g/L	3.41 g/L	.674
D‐dimer	0.28 mg/L	1.13 mg/L	<.001
FDP	3.49 μg/mL	8.26 ug/mL	<.001

Abbreviations: APTT: Activated partial thromboplastin time; FDP: Fibrin degradation products; FIB: fibrinofen; INR: International normalized ratio; PT: Prothrombin time; TT: Thrombin time.

### 3.4 Results of neurological evaluation

As for neurological function, the nonsevere group had significant lower scores than the severe group for the Framingham Stroke Risk Profile (2.31 ± 1.04 versus 8.72 ± 2.91; *p* <.05). Both the males and females in the severe patient group were found to be at a medium risk of stroke (range 6% to 9%), which was much higher than the risk rate in the nonsevere group (<5%). Compared to the nonsevere group, similar results were observed for those critically ill patients with respect to the Glasgow Coma Scale scores, with those patients who had more severe COVID‐19 having increased scores compared with the nonsevere patients (14.3 ± 0.5 versus 13.9 ± 0.4; *p* <.05).

### 3.5 Subgroup analysis

Though there were only few patients who had a medical history or new condition of stroke, a subgroup analysis was performed for these patients. The results showed that compared to nonstroke patients, those who had experienced a stroke had an increased white blood cell and neutrophil count, along with decreased lymphocyte count in routine laboratory tests (*p* <.05). As for coagulation functions, only D‐dimer showed significantly higher values in stroke patients compared to nonstroke patients (*p* <.05).

### 3.6 Correlation analysis

Correlation analyses performed between the Framingham Stroke Risk Profile score and the demographic and clinical characteristics demonstrated that there were positive associations between stroke risk scores and age in both groups (*p* <.05).

With respect to the laboratory tests, elevated white blood cell and neutrophil values were positively correlated with increased Framingham Stroke Risk Scores in critically ill patients (*p* <.05). Similar positive associations were noticed between interleukin‐6 and the Framingham Stroke Risk Profile scores (*p* <.05), while white blood cell and neutrophil values were negatively correlated with the Glasgow Coma Scale scores in critically ill patients (*p* <.05). Lymphocyte count was negatively associated with the Framingham Stroke Risk Profile scores (*p* <.05). There were no correlations within the nonsevere group.

With respect to coagulation indexes, D‐dimer and fibrin degradation products were closely correlated with both stroke risk scores (positive) and the Glasgow Coma Scale scores (negative) in severe patients (*p* <.05). No correlations were noticed in nonsevere group.

## 4 DISCUSSION

COVID‐19 is highly contagious, causing serious social and medical issues. Critically ill COVID‐19 populations with cerebrovascular disease are at risk. In our study, we evaluated 193 patients. The demographic results showed there were more male patients than females with a mean age of around 50 years. Most critically ill patients were elderly people with a mean age of 59.7 years, which is significantly older than those not admitted to the ICU. In terms of comorbidity, patients in the severe group had more underlying diseases, especially cardio‐cerebrovascular diseases like hypertension and stroke, while both severe and nonsevere symptoms were more often noticed in aged people. These demographic findings were consistent with previous reports (Huang et al., 2020; Wang, Hu, et al., 2020), suggesting that age and comorbidities were associated with the severity of illness and that individuals with a history of cerebrovascular disease may be more susceptible to severe infections (Fan et al., 2020). This increased susceptibility may be further exacerbated in male patients, who have a greater risk of stroke due to social pressure, hypertension, and alcohol use; these conditions became more prominent with aging (Poorthuis et al., 2017; Sealy‐Jefferson et al., 2012). Importantly, the risk of stroke was raised in patients with severe respiratory infections; reflecting a negative and vicious cycle of pathology (Warren‐Gash et al., 2018). Among the severe group, two cases resulted in death and another 3 were transferred to ICU in a senior hospital. All of the five critically ill patients had coexisting stroke conditions, and one of the patient who passed away had acute cerebral infarction during admission in ICU. These incidents may provide evidence to suggest that the infection associated with this new coronavirus may contribute to the occurrence of cerebrovascular diseases. Conversely, they might indicate that pre‐existing stroke conditions may deteriorate pulmonary infections, which becomes a vicious cycle. Such relationships have been described in a previous report, which showed that older individuals have a greater chance of suffering from acute ischemic stroke due to the weak immune response (Zhai et al., 2020). Less than half of the patients in this study had underlying diseases and those nonsevere patients usually resulted in a fine prognosis (Guan et al., 2020); however, stroke risks were potentially high and fatal in the new coronavirus pneumonia patients, especially for those critically ill patients. It is important to identify the risk of stroke even earlier to prevent severe complications and improve recovery rate.

The most common abnormalities in routine laboratory tests were decreased lymphocytes, especially for patients in the severe group. Though still within normal ranges, our study noticed significant increases in white blood cell and neutrophil counts in the severe group compared with the nonsevere group. The abnormalities were similar to the laboratory results observed in previous studies (Huang et al., 2020; Wang, Hu, et al., 2020; Wang, Li, et al., 2020). These findings suggested that patients with COVID‐19 suffered from cellular immune deficiency. Fan's study (Fan et al., 2020) also showed that severe infection of COVID‐19 may trigger an excessive immune response. COVID‐19 infection can induce a cytokine storm that results in neutrophilia, an inflammatory response that can activate the coagulation process and potentially represent the early stages of stroke.

Both groups in current study had levels of CRP and ESR above the normal range, which was more obvious in the critically ill patients. Both CRP and ESR were inflammation‐related indicators that have been indicated to be closely related to disease severity in this new coronavirus (Bao et al., 2020; Guan et al., 2020; Wang, Li, et al., 2020). High white blood cells and neutrophils along with elevated C‐reactive protein were often noticed in the inflammatory process. Patients with COVID‐19, especially those with a history of hypertension and cerebrovascular disease, had more stroke risk factors. These risk factors are more obvious with inflammatory stimulation, inflammatory cells affect cerebral arteries and brain tissue, resulting in atherosclerosis. Cerebral circulation was interrupted, and risk of stroke was increased (Fan et al., 2020; Warren‐Gash et al., 2018). A similar situation was noticed for interleukin‐6, with elevated IL‐6 noted in new coronavirus pneumonia patients, especially those who were critically ill. IL‐6 also was considered as proinflammatory cytokines in serum, which has been shown to be closely related to pulmonary inflammation (Wong et al., 2004). Furthermore, the severe group had significantly higher levels of IL‐6 than the nonsevere group, indicating that disease severity was associated with a cytokine storm caused by reduced T cells (Bao et al., 2020; Diao et al., 2019). These findings were in accordance with previous reports (Bao et al., 2020; Chen, Wu, et al., 2020; Wang, Li, et al., 2020) that show a combined evaluation of IL‐6, ESR, and CRP is a better predictor of disease prognosis. A previous study (Kerr et al., 2001) has shown that cytokines, like IL‐6, can contribute to thromboembolic events and plaque disruption, which is the key promoter of ischemic stroke.

In our study, platelet count was within the normal range for both groups, however, severe patients demonstrated higher platelet count, which is consistent with previous studies (Chen, Wu, et al., 2020; Huang et al., 2020). While other studies (Guan et al., 2020; Wang, Hu, et al., 2020) showed opposite results, indicating decreased platelet count in patients admitted to the ICU. Changes in platelet count were not significantly different in these studies, indicating that platelet counts may be limited for evaluating coagulation function and severity of COVID‐19. As for coagulation function, higher values were revealed in the severe group compared with the nonsevere group for most parameters on admission, reflecting a hypercoagulable state of blood. Infection of COVID‐19 led to aggregation of platelet and activation of the coagulation system (Violi et al., 2020), which is in accordance with the results of our study. During the early stages of stroke, platelet count is decreased because platelets were over consumed and the coagulation process was activated. With disease progression, a large volume of newly formed platelets was evident in the serum, which resulted in a hypercoagulable state. Reduction of platelet count in critically ill patients indicated thrombocytopenia is more frequent in this population. Especially for D‐dimer, which was an elementary indicator of severity in COVID‐19 patients (Guan et al., 2020; Huang et al., 2020; Violi et al., 2020; Wang, Hu, et al., 2020). Around 5.7% of critically ill patients with COVID‐19 suffered from acute cerebrovascular disease (Mao et al., 2020). Severe patients usually had coagulopathy with high D‐dimer and elevated prothrombin time, which is a result of endothelial dysfunction caused by an inflammatory response. The level of D‐dimer in our study was markedly elevated in the severe group. This may be explained by the notion that the critically ill patients were often in a state of hypoxia, due to severe pulmonary infections. Low blood oxygen will cause an inflammatory process leading to endothelial damage and slowed blood flow. All these resulted in increased coagulability, which was the key risk factor of cerebrovascular disease, and increased the probability of ischemic stroke (Violi et al., 2020; Zhai et al., 2020). We also noticed that most coagulation values were within a normal range for nonsevere patients, though critically ill patients had elevated PT and INR, these two parameters were still within the normal range. As for APTT and TT, both increased significantly and were out of the normal range in critically ill patients. This finding indicated that the hypercoagulability process has begun in the critical patient group, but that it still had not reached its peak. Previous studies also pointed out that platelet activation and clotting may occur at early stage of pneumonia and hypercoagulation may lead to thromboembolic events of both the arterial and venous systems (Cangemi et al., 2014, 2016; Violi et al., 2020). Thus, the earlier we noticed the possibility of stroke during early stage of severe pneumonia, the better prevention we can undertake.

Correlations between routine blood tests and disease severity were in accord with previous studies (Bao et al., 2020; Wang, Li, et al., 2020), which showed that elevated white blood cells and neutrophil cells were evident in critically ill patients. As such, this population may be at high risk of poor outcomes, including cerebrovascular diseases like stroke. Results of neurological evaluations and correlations in current research also demonstrated that critically ill patients with this new coronavirus pneumonia have high potential risks of stroke. Though increased significantly in the severe group, most COVID‐19 cases only affected the respiratory system, unless the patients became severely ill and became unconscious due to hypoxia. This would explain why the Glasgow Coma Scale scores were high in this study. Coronavirus can be spread from the respiratory tract to the brain (Desforges et al., 2014), at the same time, procoagulant state was presented and led to large cerebral arterial thromboembolism. This was more obvious in the critically ill patients who had pre‐existing conditions (Verstrepen et al., 2020). Treatments like immunoglobulins were often utilized in COVID‐19 patients, which may increase the risk of stroke due to hyperviscosity syndrome that was characterized by hypercoagulable state due to infections and inflammatory responses (Dalakas & Clark, 2003).

Hypoxia and inflammation caused by new coronavirus can affect the ischemic cerebrovascular disease (Zhai et al., 2020). Infections of COVID‐19 caused hypoxia and inflammatory cytokines to be secreted, which interfere with cell metabolism and results in intracellular acidosis and activation of inflammatory responses. Cytokines, like IL‐6, were released as shown in our study, leading to further tissue ischemia. Similarly, inflammations can trigger atherosclerosis and unstable plaques (Hartmann et al., 2015). In vitro and in vivo studies have shown that angiotensin‐converting enzyme 2 (ACE2) can improve function of endothelial progenitor cell, protecting the brain from ischemic stroke (Chen et al., 2013, 2014). There was a decline of ACE2 expression in the lung with aging and such decrease was more obvious in males than in females (Xudong et al., 2006). This was consistent with our study that most critically ill patients with COVID‐19 were elderly people who were at high risk of stroke. Additionally, ACE2 receptors were affected by this new pneumonia, patients with hypertension may have had elevated blood pressure, which in turn increases the risk of stroke (Chan et al., 2020; Hess et al., 2020; Zhou et al., 2020).

The limitation of this study included the relatively small single‐center study design, which resulted in a small number of patients being enrolled; particularly those who were classified as critically ill. As a retrospective study that was conducted in an isolation hospital for contagious diseases, we were also lacking a group of control subjects to evaluate normal respiratory infections in a similar population of patients. This may have led to some bias being introduced into our analyses.

In conclusion, COVID‐19 may introduce increased risk of stroke, especially for those who have existing cerebrovascular disease. Stroke risk after respiratory infections is usually increased during the first week and remains raised until 4 weeks (Warren‐Gash et al., 2018). It is important to identify COVID‐19 patient with high risk of stroke, especially for those severe patients, aiming to reduce mortality of potential cerebrovascular disease and improve recovery rate. COVID‐19 affects both the respiratory and nervous system. Inflammation, hypercoagulability, and hypoxia, all these pathologic mechanisms were associated with an increased risk of stroke (Warren‐Gash et al., 2018). More attention should be paid to patients with COVID‐19, especially elderly individuals with a history of cerebrovascular disease and a higher risk of stroke. Evaluating and treating such populations are particularly important.

## CONFLICTS OF INTEREST

The authors declared no conflicts of interest to this work. All authors read and approved the final version of the manuscript.

## AUTHOR CONTRIBUTIONS

Feng Qiu contributed to conceptualization and supervision. Feng Qiu, Aiqing Zhang, and Yue Wu contributed to data curation. : Feng Qiu, Ming Ding, Aiqing Zhang, and Yue Wu contributed to formal analysis and investigation. Feng Qiu, Ming Ding, Aiqing Zhang, Yue Wu, and Guojin Xie contributed to methodology. Feng Qiu, Ming Ding, Aiqing Zhang, Yue Wu, Guojin Xie, Hui Cao, Mingyang Du, Haibo Jiang, and Shun Li contributed to Writing—original draft and Writing—review and editing.

### PEER REVIEW

The peer review history for this article is available at https://publons.com/publon/10.1002/brb3.2185.

## Data Availability

The data that support the findings of this study are available on request from the corresponding author. The data are not publicly available due to privacy or ethical restrictions.
